# Genitale Dermatosen im Fokus: Nicht jede Hautveränderung ist eine Geschlechtskrankheit

**DOI:** 10.1007/s00105-026-05652-y

**Published:** 2026-02-25

**Authors:** Damian Kostner, Caroline Possanner, Johanna Strobl

**Affiliations:** Universitätsklinik für Dermatologie, Medizinische Universität Wien, Währinger Gürtel 18–20, 1090 Wien, Österreich

**Keywords:** Chronisch entzündliche Dermatosen, Genitoanale Region, Differenzialdiagnosen, Chronic dermatitis, Genital-anal region, Differential diagnosis

## Abstract

Chronisch entzündliche Dermatosen gehören zu den häufigsten Hauterkrankungen und können auch die genitoanale Region betreffen. In diesen Lokalisationen werden sie oft spät erkannt, weisen ein variantenreiches klinisches Erscheinungsbild auf und erfordern eine sorgfältige dermatologische Abklärung. Der vorliegende Beitrag bietet einen Überblick über ausgewählte entzündliche Dermatosen mit genitoanalem Befall und legt den Schwerpunkt auf klinische Präsentationen, diagnostische Vorgehensweisen und therapeutische Optionen.

## Abstract (en)

Chronic inflammatory dermatoses are among the most common skin disorders and can also affect the genitoanal region. In these locations, they are often recognized late, display a wide range of clinical manifestations, and require thorough dermatological evaluation. This article provides an overview of selected inflammatory dermatoses involving the genitoanal region, with particular emphasis on clinical presentation, diagnostic work-up, and therapeutic approaches.

## Lichen sclerosus

Der Lichen sclerosus (LS; ehem. Lichen sclerosus et atrophicus) ist eine chronisch entzündliche Dermatose multifaktorieller Genese, deren Ätiologie bislang nicht vollständig geklärt ist. Die Prävalenz wird auf etwa 0,1–0,3 % bei beiden Geschlechtern geschätzt, jedoch bis 0,7 % bei Frauen über 65 Jahren [17]. Aufgrund unspezifischer Symptome, Tabuisierung und Scham-assoziierter Krankheitsvermeidung sowie oligosymptomatischen bis asymptomatischen Verläufen (ca. 30 % der Fälle) ist jedoch von einer deutlich höheren Dunkelziffer auszugehen.

**Abb. 1:**
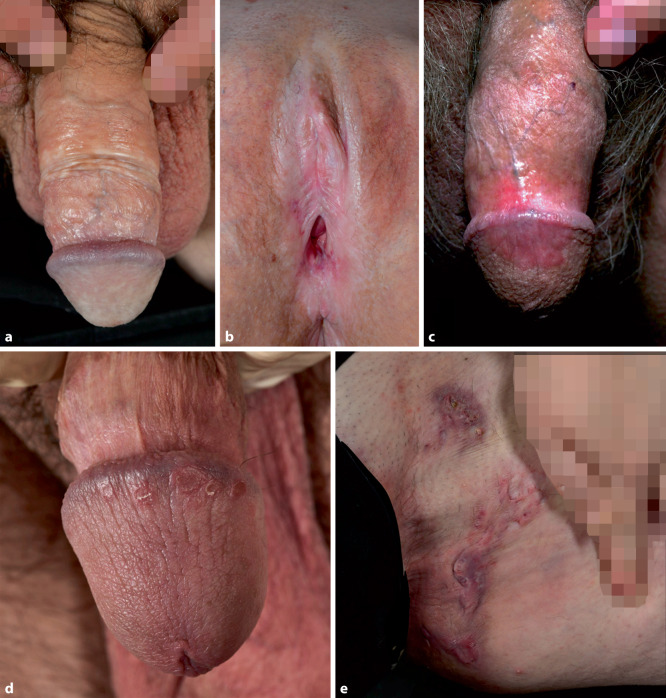
**a** Lichen sclerosus des Penis und **b** der Vulva. **c** Genitaler Lichen planus. **d** Psoriasis inversa mit erythrosquamösen Papeln der Glans penis. **e** Hidradenitis suppurativa mit inguinalen kommunizierenden Fisteln und Vernarbungen (Hurley Stadium III). (Mit freundl. Genehmigung, ©Fotolabor, Universitätsklinik für Dermatologie, Medizinische Universität Wien)

In etwa 85 % der Fälle ist der Befall auf die Genitalregion beschränkt, extragenitale Manifestationen sind selten. Bei beiden Geschlechtern kommt es neben Juckreiz häufig zu Brennen, Erythemen, Erosionen und im Verlauf zu sklerotischen Veränderungen mit Narbenbildung und Verwachsungen. Klinisch präsentiert sich der LS initial typischerweise mit starkem Pruritus und flachen, weißlich-elfenbeinfarbenen, wachsartigen Makulae, die konfluieren und zu atrophen, gefältelten oder – seltener – hyperkeratotischen Plaques übergehen können. Geschlechtsspezifisch zeigen sich bei Frauen periklitorale Schwellungen, Fissuren, Ekchymosen, und es kommt bei fortschreitendem Verlauf häufig zum Verlust der kleinen Schamlippen und der Klitorisvorhaut (Abb. 1a). Bei Männern hingegen sind v. a. Glans und Präputium betroffen (Abb. 1b), typischerweise mit porzellanartig weißlichen, sklerotischen Narben, die Phimose, Verwachsungen oder ein sklerotisches Frenulum breve begünstigen.

Bei beiden Geschlechtern können Verwachsungen und anatomische Veränderungen zu urogenitalen Symptomen und sexuellen Dysfunktionen wie Dyspareunien, schmerzhaften Erektionen und Anorgasmie führen.

Wichtig ist zu beachten, dass LS eine fakultative Präkanzerose darstellt: Bis zu 4 % der Betroffenen entwickeln nichtmelanozytäre Karzinome der Haut, überwiegend Plattenepithelkarzinome [16]. Eine frühzeitige Diagnose und adäquate Therapie sind daher essenziell. Die Diagnose des LS erfolgt meist klinisch und kann in unklaren Fällen oder bei Verdacht auf Malignität durch eine Biopsie ergänzt werden. Differenzialdiagnostisch kommen der Lichen planus – hier v. a. die erosive Form –, Ekzeme sowie in bestimmten Fällen auch eine genitale Vitiligo infrage.

Lichen sclerosus stellt eine fakultative Präkanzerose dar

Ziel der Behandlung ist die Kontrolle der Erkrankung und Linderung der Symptome, insbesondere Heilung von Fissuren und Erosionen und Reduktion der Sklerose. Erstlinientherapie sind hochpotente topische Glukokortikoide (Klasse III–IV, Clobetasolpropionat 0,05 % Salbe oder Mometasonfuroat 0,1 % Salbe), die 1‑ bis 2‑mal täglich über bis zu 3 Monate aufgetragen werden, gefolgt von einer Erhaltungsphase in längeren Intervallen zur Rezidivprophylaxe. Ein Standardschema existiert gemäß Leitlinie nicht. Bei Unverträglichkeit oder unzureichendem Ansprechen können (additiv) topische Calcineurininhibitoren (Tacrolimus oder Pimecrolimus) „off-label“ eingesetzt werden. Begleitend werden 2‑mal täglich topische Emollienzien zur Basispflege und die Vermeidung von Triggerfaktoren (Reibung, Nässe) dringend empfohlen. In schweren therapierefraktären Fällen kann unter Beachtung der Teratogenität eine Therapie mit systemischen Retinoiden (Acitretin 20–30 mg) oder eine systemische Immunsuppression initiiert werden (Methotrexat 10–15 mg/Woche), wobei die Evidenzlage für Systemtherapien insgesamt schwach ist. Eine frühzeitige operative Sanierung mittels Zirkumzision ist bei männlichen Patienten mit Befall des Präputiums indiziert, bei Verengungen der Harnröhre bzw. Meatusstenose die Urethroplastik und Meatusplastik. Im Spätstadium stellt auch bei Frauen die chirurgische Sanierung eine weitere Behandlungsoption dar, die bei Synechien der Labien oder Verengung des Orificium externum der Urethra indiziert sein kann [23].

## Lichen planus mucosae

Der Lichen planus (LP) ist die prototypische lichenoide Dermatitis und eine nichtinfektiöse, entzündliche Erkrankung von Haut und Schleimhäuten. Klinisch dominieren starker Pruritus und charakteristische, flache, polygonale Papeln. Die Prävalenz beträgt etwa 1 %, Frauen sind häufiger betroffen als Männer [5]. Am häufigsten manifestiert sich der LP an den Schleimhäuten (auch Lichen planus mucosae), v. a. enoral, jedoch tritt ein genitaler Befall nicht selten auf [3].

Bei Männern zeigt sich der genitale LP überwiegend in der klassischen Form mit rosafarbenen bis violetten glänzenden Papeln an Eichel und Sulcus coronarius (Abb. 1c). Erosive Veränderungen sind seltener, können jedoch Schmerzen, Dysurie und Vernarbungen verursachen. Bei Frauen dominiert hingegen die erosive Form mit glasigen, erythematösen bis violetten Läsionen mit hyperkeratotischem Rand, typischerweise an Vestibulum, kleinen Schamlippen und Vagina. Im Verlauf können Vernarbungen, Synechien und Einengungen des Scheideneingangs entstehen, was Dyspareunie und sexuelle Dysfunktion zur Folge haben kann. Seltener treten papulosquamöse oder hypertrophe Formen auf.

Chronisch erosive oder hypertrophe Verläufe können, ähnlich wie beim LS, zu Narbenbildung, anatomischen Veränderungen und funktionellen Einschränkungen führen. Der LP mucosae gilt, im Gegensatz zur rein kutanen Form, als Präkanzerose mit einem Lebenszeitrisiko von etwa 2 % für die Entwicklung von Plattenepithelkarzinomen, was ein langfristiges Monitoring der Betroffenen erforderlich macht [7]. Die Diagnose des LP wird primär klinisch gestellt, kann jedoch histologisch abgesichert werden. Vor allem bei der erosiven Form sollten differenzialdiagnostisch aphthöse Erkrankungen, bullöse Autoimmundermatosen (Pemphigus vulgaris, Schleimhautpemphigoid) sowie infektiöse Ursachen wie genitale Herpesinfektionen und Kandidosen sowie Syphilis, Ulcus molle und Lymphogranuloma venereum ausgeschlossen werden.

Die Diagnose des Lichen planus mucosae wird primär klinisch gestellt

Therapeutisch stehen beim genitalen LP die Linderung der Symptome und die Eindämmung der Entzündung im Vordergrund. Mittel der ersten Wahl sind mittel- bis hochpotente topische Glukokortikoide, die über mehrere Wochen täglich und anschließend in Erhaltungsintervallen appliziert werden. Bei unzureichendem Ansprechen oder Unverträglichkeiten können topische Calcineurininhibitoren („off-label“) eingesetzt werden. In schweren oder therapierefraktären Fällen kann eine systemische Therapie mit Kortikosteroiden, Retinoiden oder eine immunsuppressive Therapie (Cyclosporin 3–10 mg/kg/Tag; „second line“: Azathioprin, Hydroxychloroquin, Methotrexat, Mycophenolatmofetil) erforderlich sein. Ergänzend sind in ausgewählten Situationen chirurgische Maßnahmen indiziert, insbesondere bei funktionellen Einschränkungen [9, 24].

## Psoriasis inversa

Die Psoriasis vulgaris ist eine chronisch entzündliche Hauterkrankung, die etwa 2 % der europäischen und nordamerikanischen Bevölkerung betrifft und meist in ihrer typischen Form, der chronischen Plaquepsoriasis, auftritt. Die Psoriasis inversa (PI) stellt dabei keine eigenständige Entität dar, sondern ein lokalisiertes Auftreten der Psoriasis vorwiegend oder ausschließlich in intertriginösen Arealen. Die Prävalenz der PI bei Personen mit Psoriasis wird je nach Studie mit bis zu 30 % angegeben [12].

Die Plaquepsoriasis präsentiert sich typischerweise mit gut abgrenzbaren, erythematösen, silbrig schuppenden Plaques, die bevorzugt an Ellenbogen, Knien, Sakralregion und Kopfhaut auftreten. Im Gegensatz zeigen sich die Plaques im Bereich der intertriginösen Areale (axillär, submammär, Labien, Skrotum, Analfalte) klinisch weniger induriert, nässend, mit fehlender oder nur minimaler oberflächlicher Schuppung und häufig mazeriert (Abb. 1d). Die Diagnose der PI erfolgt in erster Linie klinisch und histologisch, zudem kann aber eine mikrobielle Abklärung notwendig sein, um klinisch ähnliche Differenzialdiagnosen (Tinea corporis, Kandidose, Intertrigo) auszuschließen und bakterielle Superinfektionen bei PI gezielt zu behandeln.

Die Behandlung der PI ist aufgrund des warmen, feuchten Milieus der Hautfalten anspruchsvoll. Zwar erleichtert die geringere Hornschicht intertriginöser Areale die Penetration topischer Medikamente, gleichzeitig steigt jedoch das Risiko für unerwünschte Nebenwirkungen wie Irritationen und Atrophie, insbesondere bei Anwendung hochpotenter Kortikosteroide. Topische Therapien umfassen daher niedrig- bis mittelpotente Kortikosteroide, Calcipotriol und „off-label“ Calcineurininhibitoren, die sich bei der PI als wirksam erwiesen haben und keine Atrophie verursachen. Ergänzend empfehlen sich Emollienzien, Zinksalben, Trockenhalten betroffener Regionen sowie bei Bedarf antibiotische oder antimykotische Maßnahmen.

Bei therapieresistenter PI sollte frühzeitig eine systemische Behandlung mit konventionellen Systemtherapeutika (Fumarate, Methotrexat) oder Biologika gemäß Leitlinien der Plaquepsoriasis erwogen werden. Eine Beteiligung des Genitalbereichs stellt hier ein sog. Upgrade-Kriterium dar, aufgrund dessen bereits bei geringem BSA-Befall eine Systemtherapie indiziert ist [25]. Auch wenn die direkte Studienlage in Bezug zur PI schwach ist, zeigen kleinere Fallserien eine vergleichbare Ansprechrate zur Plaquepsoriasis [20].

## Hidradenitis suppurativa

Die Hidradenitis suppurativa (HS) ist eine chronisch rezidivierende, entzündliche Hauterkrankung multifaktorieller Genese. Pathophysiologisch steht eine follikuläre Okklusion im Vordergrund, begünstigt durch genetische Prädisposition, immunologische Dysregulation sowie verschiedene äußere Einflüsse. Die enge Assoziation mit Adipositas und Nikotinkonsum unterstreicht die Bedeutung externer Risikofaktoren [1, 21]. Betroffene leiden häufig unter erheblichem psychosozialem Leidensdruck, und es bestehen Assoziationen mit multiplen Komorbiditäten wie Diabetes mellitus, Atherosklerose und chronisch entzündlichen Darmerkrankungen.

Die Assoziation mit Adipositas und Nikotinkonsum unterstreicht die Bedeutung externer Risikofaktoren

Klinisch manifestiert sich die Erkrankung durch schmerzhafte, entzündliche Knoten, Abszesse und Fistelgänge, die bevorzugt in intertriginösen und apokrindrüsenreichen Arealen auftreten, insbesondere Axillae, Inguinal- und Perianalregion, submammär sowie im Gesäßbereich (Abb. 1e). Die chronische Inflammation kann zu Erosionen, Ulzerationen, fibrotischen Plaques und hypertrophen Narben führen sowie bei längerem Krankheitsverlauf zur Bildung von epithelialisierten Tunneln.

Die Diagnose wird klinisch gestellt und anhand der International Hidradenitis Suppurativa Severity Score System (IHS)4-Kriterien (Tab. 1) in milde, moderate/mittelschwere und schwere Verlaufsformen eingestuft. Differenzialdiagnostisch sollten bei untypischem Erscheinungsbild Furunkulosen sowie abszessbildende Infektionen anderer Genese ausgeschlossen werden (z. B. kutane MRSA-Infektionen oder atypische Mykobakteriosen). Bei bestehender Grunderkrankung ist zudem an eine – gegebenenfalls fistulierende – kutane Manifestation eines Morbus Crohn zu denken.

**Tab. 1:** Internationales Bewertungssystem für den Schweregrad der Hidradenitis suppurativa (IHS4)

IHS4 Punktesystem		
	Anzahl der Knoten × 1	
*+*	Anzahl der Abszesse × 2	
*+*	Anzahl der Fisteln × 4	
*=*	Gesamtpunktezahl:	
	*≤* *3: milde HS*	
	*4–10: mittelschwere HS*	
	*≥* *11: schwere HS*	

Die Therapie richtet sich nach Schwere- und Entzündungsgrad, Grundlage ist jedoch stets die Empfehlung zur Gewichtsreduktion und Nikotinkarenz, sofern zutreffend. Bei milder aktiver HS (IHS4 1–3) kommen topische Antibiotika wie Clindamycin Gel 2-mal täglich sowie topische Antiseptika zum Einsatz. Bei moderat bis schwerer aktiver HS (IHS4 ≥ 4) ist eine systemische Therapie indiziert. Initial wird eine orale antibiotische Therapie über maximal 12 Wochen mit Doxycyclin 2 × 100 mg/d oder alternativ eine Kombinationstherapie mit Clindamycin 2 × 300 mg/d und Rifampicin 2 × 300 mg/d empfohlen. Bei therapierefraktärer oder schwerer HS stehen zudem Biologika zur Verfügung, insbesondere die aus der Psoriasistherapie bekannten TNF-α-Inhibitoren, Interleukin(IL)-17A-Inhibitoren sowie IL-17A/F-Inhibitoren, die in höherer Frequenz oder Dosierung als für die Plaquepsoriasis verabreicht werden. Weitere Substanzen befinden sich in klinischer Prüfung. In inaktiven (nichtentzündlichen) Intervallen und/oder bei ausgeprägter Fistelbildung ist eine chirurgische Sanierung in der Regel indiziert, um persistierende Läsionen zu entfernen [22].

**Tab. 2:** Diagnosekriterien des Morbus Behçet nach ISG und nach ICBD

ISG-Kriterien (1990)		ICBD-Kriterien (2014)	
Rezidivierende orale Aphthose	(≥ 3 Episoden innerhalb von 12 Monaten)	Orale Aphthose	2 Punkte
*Plus mindestens 2 der folgenden Kriterien:*		Genitale Ulzera	2 Punkte
Rezidivierende genitale Ulzera	Narbenbildend	Augenbeteiligung	2 Punkte
Augenbeteiligung	Uveitis anterior/posterior, Retinitis, Gefäßveränderungen	Hautläsionen	1 Punkt
Hautläsionen	Erythema nodosum, papulopustulöse oder akneiforme Läsionen	Neurologische Manifestation	1 Punkt
Positiver Pathergietest	Nach 24–48 h papulopustulöse Reaktion auf sterile Nadelstichung	Vaskuläre Manifestation	1 Punkt
–		Positiver Pathergietest	1 Punkt (optional)
		*≥* *4 Punkte* *→* *Diagnose Morbus Behçet wahrscheinlich*	

## Ekzeme

Genitale Ekzeme stellen einen häufigen dermatologischen Vorstellungsgrund dar, wobei insbesondere die atopische Dermatitis (AD) und die allergische oder irritativ-toxische Kontaktdermatitis differenzialdiagnostisch von Relevanz sind.

**Abb. 2:**
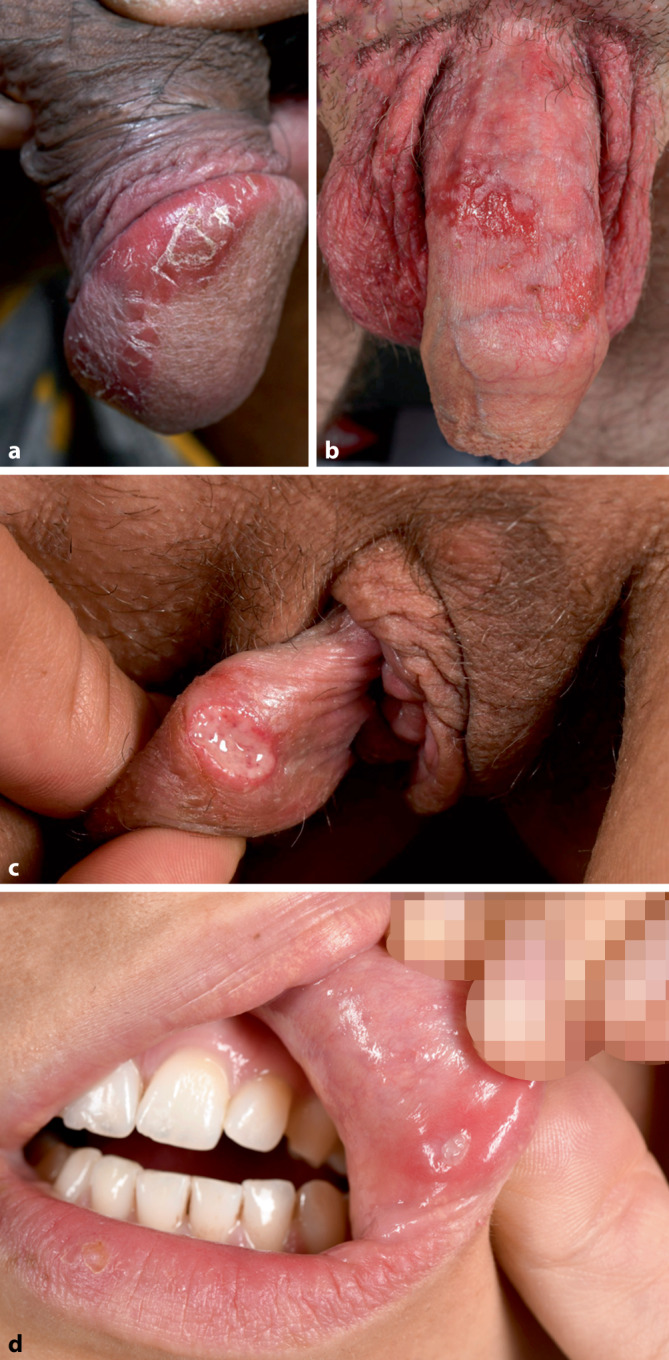
**a** Genitales Ekzem bei atopischer Dermatitis. **b** Erosive Kontaktdermatitis nach Anwendung von Podophyllotoxin. **c** Genitalulkus und **d** orale Aphthe, beides bei Morbus Behçet. (Mit freundl. Genehmigung, ©Fotolabor, Universitätsklinik für Dermatologie, Medizinische Universität Wien)

Die AD ist eine chronisch entzündliche, immunvermittelte Dermatose mit multifaktorieller Pathogenese, in deren Entstehung genetische Prädisposition, epidermale Barrierestörung, immunologische Dysregulation sowie Umweltfaktoren involviert sind. Die Prävalenz zeigt sich in den letzten Jahrzehnten steigend und beträgt bis zu 20 % der Kinder und etwa 10 % der Erwachsenen [6]. Klinisch imponieren stark juckende, erythematös-ödematöse, unscharf begrenzte Ekzemherde, die Papeln, Vesikel, Krusten- oder Schuppenbildung aufweisen können und bei chronischem Kratzen exkoriiert oder lichenifiziert sein können. Typischerweise manifestieren sich die Läsionen in den klassischen Prädilektionsarealen (Kopf-Hals-Region, Beugen, Hände und Füße); eine Genitalbeteiligung ist jedoch ebenfalls möglich (Abb. 2a).

Die Kontaktdermatitis hingegen resultiert aus exogenen Noxen, entweder im Sinne einer irritativen/toxischen oder einer allergischen Reaktion, und präsentiert sich mit lokalisierten, häufig pruriginösen Ekzemen, die bei entsprechender Exposition auch genital auftreten können (Abb. 2b).

Ebenfalls dem Formenkreis der Ekzeme zuzuordnen ist der perianale Lichen simplex chronicus als Sonderform des Lichen simplex chronicus. Dabei handelt es sich um sekundäre Hautveränderungen, die infolge rezidivierender mechanischer Manipulation bei chronischem Pruritus im Sinne eines Juck-Kratz-Teufelskreises entstehen. Klinisch manifestieren sich diese meist als perianale, scharf begrenzte, ekzematöse bis lichenoide Plaques, bei längerem Bestehen auch als hyperkeratotische oder noduläre, prurigoartige Veränderungen. Die Ursachen des perianalen Pruritus sind vielfältig, häufig liegen jedoch irritative Intimpflege, Hämorrhoidalleiden, feuchtes Milieu oder eine atopische Diathese zugrunde.

Bei Verdacht auf eine allergische Kontaktdermatitis kann eine allergologische Testung sinnvoll sein

Die Diagnosestellung erfolgt primär klinisch und mittels bakteriologischer Diagnostik zum Ausschluss allfälliger Superinfektionen. Bei Verdacht auf eine allergische Kontaktdermatitis kann eine allergologische Testung (Epikutantests) sinnvoll sein. Therapeutisch stehen die Elimination exogener Triggerfaktoren (z. B. Desinfektionsmittel, Duftstoffe, Latex, lokale chemische Kontrazeptiva) sowie eine konsequente Basistherapie mit Emollienzien im Vordergrund. Leichte bis moderate Läsionen sprechen in der Regel auf eine kurzzeitige Behandlung mit topischen Glukokortikosteroiden oder alternativ Calcineurininhibitoren an. Systemische Therapien sind bei isoliert genitaler Manifestation in der Regel nicht erforderlich und sollten lediglich in ausgewählten Einzelfällen erwogen werden [26, 27]. Beim perianalen Lichen simplex chronicus steht die konsequente Vermeidung des Kratzens im Vordergrund. Kurzfristige topische Kortikosteroidtherapien können dabei helfen, den Juck-Kratz-Teufelskreis zu durchbrechen. Therapierbare Ursachen des Pruritus (z. B. Hämorrhoiden) sollten gezielt mitbehandelt werden.

## Fixes Arzneimittelexanthem

Hautreaktionen zählen zu den häufigsten unerwünschten Arzneimittelwirkungen. Eine besondere Form stellt das fixe Arzneimittelexanthem (AZME) dar, eine Typ-IV-Hypersensitivitätsreaktion, die typischerweise innerhalb von 48 h nach Exposition auftritt und sowohl Haut als auch (Genital‑)Schleimhäute betreffen kann. Klinisch zeigen sich solitäre scharf begrenzte, runde bis ovale erythematöse oder livide Makulae, häufig mit zentraler Hyperpigmentierung, seltener mit bullöser Komponente oder Streuung (multilokuläres fixes AZME). Als prototypische Reaktion hautresidenter T‑Zellen rezidiviert das fixe AZME bei Re-Exposition charakteristischerweise in loco. Eine genitale Beteiligung (Mukosa der Glans penis oder Vulva) ist dabei häufig, v. a. bei Männern, und wird in der Literatur in bis zu 30 % der Fälle beschrieben. Zu den häufigsten Auslösern eines fixen AZME zählen Analgetika (insbesondere NSAR und Paracetamol) sowie Antibiotika (v. a. Cotrimoxazol) [4, 11].

Die Diagnose erfolgt klinisch und in Zusammenschau mit der Anamnese. Gelegentlich kann eine allergologische In-loco-Patch-Testung ein positives Ergebnis erzielen. Therapeutisch stehen die Identifikation und das Absetzen des auslösenden Medikaments im Vordergrund. Bei milden bis moderaten Verläufen kann eine kurzzeitige topische Therapie mit Glukokortikoiden sowie ggf. die Gabe von Antihistaminika zur Symptomlinderung erfolgen [2, 28].

## Morbus Behçet

Der Morbus Behçet ist eine inflammatorische Multisystemerkrankung unklarer Ätiologie mit sowohl autoinflammatorischen als auch autoimmunologischen Merkmalen. Pathogenetisch zählt sie zu den MHC-I-assoziierten Erkrankungen, wobei insbesondere HLA-B51 und HLA-A26 prädisponierend wirken. Histologisch findet sich typischerweise eine leukozytoklastische Vaskulitis mit neutrophilen Infiltraten, Endothelschädigung und fibrinoider Nekrose. Die Erkrankung tritt weltweit auf, zeigt jedoch eine deutlich höhere Prävalenz entlang der historischen „Seidenstraße“, insbesondere in der Türkei, im Nahen Osten und in Fernost-Asien, mit Raten von bis zu 300 pro 100.000 Einwohner, während sie in Mitteleuropa mit weniger als 1 pro 100.000 deutlich seltener ist [10, 19].

Klinisch zeigt sich der Morbus Behçet mit rezidivierenden, schmerzhaften oralen und genitalen Aphthen sowie kutanen Läsionen als Leitsymptom (Abb. 2**c und d**). Die Aphthen sind oft multipel, mit grauweißer Pseudomembran und von einem erythematösem Randsaum begrenzt. Kleine Läsionen heilen narbenlos ab, größere Aphthen können ulzerieren und narbig abheilen.

Die genitalen Ulzera stellen nach den oralen Aphthen das zweithäufigste klinische Symptom dar und präsentieren sich als ausgestanzte, stark schmerzhafte Ulzera mit fibrinösem Exsudat an der Basis, die eine starke Ähnlichkeit zu syphilitischen Primäraffekten aufweisen können. Bei Männern finden sie sich überwiegend skrotal, während sie bei Frauen häufiger an den Labien lokalisiert sind.

Der Morbus Behçet ist eine inflammatorische Multisystemerkrankung

Zusätzlich können gastrointestinale, vaskuläre, neurologische, artikuläre und okuläre Manifestationen (Retinitis, Uveitis) auftreten, die den multisystemischen Charakter der Erkrankung unterstreichen.

Da pathognomonische Labor- oder Bildgebungsbefunde fehlen, erfolgt die Diagnosestellung klinisch anhand internationaler Kriterien (Tab. 2). Unterstützend können ein positiver Pathergietest sowie die Bestimmung prädisponierender HLA-Antigene (z. B. HLA-B51) herangezogen werden [14]. Differenzialdiagnostisch kommen insbesondere eine rezidivierende Aphthose sowie ein Morbus Crohn mit oraler oder genitaler Beteiligung in Betracht, ferner auch blasenbildende Autoimmundermatosen. Bei jungen Patientinnen mit akut auftretenden, schmerzhaften genitalen Ulzera ist das Ulcus vulvae acutum (Lipschütz-Ulkus) als wichtige Differenzialdiagnose zu bedenken. Vor allem bei Erstmanifestationen oder gleichzeitig bestehenden systemischen Symptomen sollten eine Herpes-simplex-Erstinfektionen, Medikamentenreaktionen (Stevens-Johnson-Syndrom) oder eine reaktive Arthritis ausgeschlossen werden.

Die Therapie richtet sich nach dem Schweregrad und Organbefall und erfolgt gemäß den aktuellen European Alliance of Associations for Rheumatology (EULAR)-Empfehlungen. Leichte mukokutane Manifestationen werden in der Regel topisch mit Antiseptika, lokalanästhetischen Externa oder lokalen Glukokortikoiden behandelt. Bei schwerer Symptomatik können kurzzeitig systemische Glukokortikoide angewandt werden. Bei moderaten bis schweren Verlaufsformen oder häufigen Rezidiven ist eine systemische immunsuppressive/-modulatorische Therapie indiziert, deren Auswahl sich nach den Leitsymptomen, Patientenmerkmalen und betroffenen Organsystemen richtet. Therapeutische Optionen umfassen Colchicin, Azathioprin, Thalidomid, Interferon‑α, Dapson, TNF-α-Inhibitoren sowie Phosphodiesterase-4-Inhibitoren (z. B. Apremilast) [8].

## Plasmazellbalanitis (Zoon-Balanitis)

**Abb. 3:**
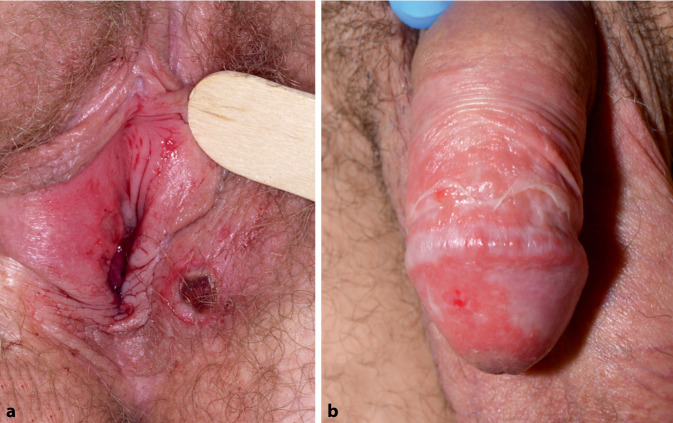
**a** Ulcus vulvae acutum Lipschütz. **b** Plasmazellbalanitis. (Mit freundl. Genehmigung, ©Fotolabor, Universitätsklinik für Dermatologie, Medizinische Universität Wien)

Die Plasmazellbalanitis (PZB) ist eine chronische, idiopathische, reaktive Balanoposthitis, die überwiegend bei unbeschnittenen älteren Männern auftritt und vermutlich sekundär infolge einer Vorhautdysfunktion entsteht, häufig im Zusammenhang mit Mikrotraumata und unzureichender Hygiene. Eine Dysfunktion des Präputiums begünstigt die Retention von Wärme, Smegma und Feuchtigkeit und erhöht dadurch die Anfälligkeit für sekundäre Infektionen. Typischerweise manifestiert sich die PZB an Vorhaut und Eichel als symmetrische, scharf begrenzte, erythematöse, glänzende Plaques mit feinen roten Punkten, den charakteristischen „Cayennepfefferflecken“ (Abb. 3b).

Im Gegensatz zu anderen entzündlichen genitalen Dermatosen gilt die PZB nicht als Präkanzerose, sollte jedoch histologisch abgeklärt werden, um Malignome mit ähnlichem Erscheinungsbild, insbesondere den extramammären Morbus Paget oder die Erythroplasie de Queyrat, auszuschließen [13].

Therapeutisch kommen Behandlungen mit Calcineurininhibitoren, Kortikosteroiden oder 2 % Mupirocin-Salbe über mehrere Wochen zur Anwendung. Chronisch rezidivierende Verläufe sind jedoch häufig. In diesen Fällen bleibt die Zirkumzision die Therapie der Wahl, die in der Regel zu einer vollständigen und anhaltenden Remission führt [13]. Ergänzend ist stets eine Schulung zur genitalen Hygiene empfehlenswert.

Wenngleich deutlich seltener, existiert ein analoges Krankheitsbild bei Frauen, bekannt als Plasmazellvulvitis oder Zoon-Vulvitis. Klinisch präsentiert sie sich mit rötlich-braunen, glänzenden Plaques an den Labien oder der Klitoris sowie ebenfalls fein punktförmigen Erythemen (Cayennepfefferflecken). Diagnostik und Lokaltherapie entsprechen weitgehend jenen der Plasmazellbalanitis.

## Ulcus vulvae acutum (Lipschütz-Ulkus)

Das Ulcus vulvae acutum ist eine sehr seltene, vermutlich unterdiagnostizierte Erkrankung, die durch das plötzliche Auftreten schmerzhafter, nekrotischer Ulzera der Vulva charakterisiert ist. Betroffen sind nahezu ausschließlich (> 90 %) junge Frauen unter 20 Jahren, typischerweise unabhängig von vorangegangenem Sexualkontakt [18].

Die Ätiologie ist bislang nicht vollständig geklärt. Vermutet wird eine postinfektiöse Hypersensibilitäts- oder immunvermittelte Reaktion, häufig im Anschluss an eine Primärinfektion mit Epstein-Barr-Virus (EBV). Auch andere Infektionserreger wie u. a. Zytomegalievirus (CMV), *Mycoplasma pneumoniae*, Mumpsvirus wurden im Zusammenhang beschrieben.

Das Ulcus vulvae acutum kann solitär oder multipel auftreten und ist meist an den Labiae minorae lokalisiert, seltener an den großen Labien (Abb. 3a) oder dem Perineum. Charakteristisch sind dabei stark schmerzhafte, scharf begrenzte, tief ulzerierte und durch fibrinöse Beläge oder gräuliches Exsudat gekennzeichnete Läsionen.

Die Diagnose wird klinisch und als Ausschlussdiagnose gestellt, nachdem sexuell übertragbare Infektionen (insbesondere Herpesinfektion, Ulcus durum und Ulcus molle) sowie andere ulzerative Erkrankungen wie der Morbus Behçet, aphthöse Ulzera, traumatische Läsionen und medikamenteninduzierte Reaktionen ausgeschlossen wurden.

Das Ulcus vulvae acutum ist in der Regel selbstlimitierend und heilt innerhalb von 2 bis 3 Wochen spontan ab. Rezidive sind selten, jedoch in Zusammenhang mit erneuten Infektionen beschrieben. Die Therapie ist daher vorwiegend symptomatisch und zielt auf Schmerzlinderung sowie die Vermeidung sekundärer Infektionen ab. Topische Anästhetika und orale Analgetika können zur Schmerzkontrolle eingesetzt werden. Topische Glukokortikoide können die Abheilung beschleunigen – systemische Glukokortikoide sollten nur in ausgewählten Fällen in Betracht gezogen werden [15]. Antibiotika sind nur bei nachgewiesenen bakteriellen Sekundärinfektionen indiziert und spielen in der Primärtherapie keine Rolle.

## Fazit für die Praxis

- Genitoanale Dermatosen sind häufig, werden jedoch aufgrund unspezifischer Symptome und Tabuisierung oft spät diagnostiziert.
- Es sollten eine sorgfältige Inspektion, vollständige Anamnese und frühzeitige Biopsie bei therapierefraktären Läsionen erfolgen.
- Lichen sclerosus und Lichen planus sind wichtige Differenzialdiagnosen mit dem Risiko funktioneller Einschränkungen und (bei LS) potenzieller maligner Entartung: Regelmäßige Kontrollen sind erforderlich.
- Psoriasis inversa benötigt eine differenzierte topische Therapie; eine frühe Systemtherapie sollte erwogen werden (Genitalbefall als Schweregrad-Upgrade).
- Die Hidradenitis suppurativa sollte früh erkannt und nach IHS4 eingestuft werden. Lifestyle-Modifikation, Antibiotika und Biologika sollten leitliniengerecht eingesetzt werden.
- Ekzeme und Kontaktdermatitiden bleiben häufig: Konsequente Triggerelimination und Basistherapie sind zentral.
- Ulzerierende Erkrankungen (Morbus Behçet, Lipschütz-Ulkus) erfordern ein systematisches Ausschlussverfahren und u. U. eine interdisziplinäre Betreuung.
- Nicht stigmatisierende Kommunikation und ausführliche Patientenaufklärung verbessern Adhärenz und Langzeitverlauf.
